# Role of the Intracellular Nucleoside Transporter ENT3 in Transmitter and High K^+^ Stimulation of Astrocytic ATP Release Investigated Using siRNA Against ENT3

**DOI:** 10.1177/1759091414543439

**Published:** 2014-07-22

**Authors:** Dan Song, Junnan Xu, Qiufang Bai, Liping Cai, Leif Hertz, Liang Peng

**Affiliations:** 1 Laboratory of Brain Metabolic Diseases, Institute of Metabolic Disease Research and Drug Development, China Medical University, Shenyang, P. R. China; 2 Laboratory of Molecular Biology, Liaoning University of Traditional Chinese Medicine, Shenyang, P. R. China

**Keywords:** adenosine, ATP release, ENT3, gliotransmitter, glutamate, SLC17A9

## Abstract

This study investigates the role of the intracellular adenosine transporter equilibrative nucleoside transporter 3 (ENT3) in stimulated release of the gliotransmitter adenosine triphosphate (ATP) from astrocytes. Within the past 20 years, our understanding of the importance of astrocytic handling of adenosine, its phosphorylation to ATP, and release of astrocytic ATP as an important transmitter has become greatly expanded. A recent demonstration that the mainly intracellular nucleoside transporter ENT3 shows much higher expression in freshly isolated astrocytes than in a corresponding neuronal preparation leads to the suggestion that it was important for the synthesis of gliotransmitter ATP from adenosine. This would be consistent with a previously noted delay in transmitter release of ATP in astrocytes but not in neurons. The present study has confirmed and quantitated stimulated ATP release in response to glutamate, adenosine, or an elevated K^+^ concentration from well-differentiated astrocyte cultures, measured by a luciferin–luciferase reaction. It showed that the stimulated ATP release was abolished by downregulation of ENT3 with small interfering RNA (siRNA), regardless of the stimulus. The concept that transmitter ATP in mature astrocytes is synthesized directly from adenosine prior to release is supported by the postnatal development of the expression of the vesicular transporter SLC17A9 in astrocytes. In neurons, this transporter carries ATP into synaptic vesicles, but in astrocytes, its expression is pronounced only in immature cells and shows a rapid decline during the first 3 postnatal weeks so that it has almost disappeared at the end of the third week in well-differentiated astrocytes, where its role has probably been taken over by ENT3.

## Introduction

A multitude of authors have shown Ca^2+^-dependent release of adenosine triphosphate (ATP) from astrocytes as recently refereed by ourselves (Hertz et al., 2014). It was also mentioned that the release of ATP from cultured astrocytes triggered by receptor stimulation or Ca^2+^ uncaging occurs after a delay, with approximately only 4% of the release occurring during the first 30 s (Pryazhnikov & Khiroug, 2008), and that the time interval is even longer in brain slices (Heinrich, Andó, Túri, Rózsa, & Sperlágh, 2012). Pangrsic et al. (2007) similarly noticed a longer poststimulatory delay for the release of ATP from astrocytes than from neurons.

The released ATP must have been synthesized in the astrocytes, which cannot accumulate ATP from their surroundings. Adenosine is present in the extracellular space and can be accumulated through an equilibrative nucleoside transporter (ENT) or concentrative (CNT) nucleoside transporter (Li, Gu, Hertz, & Peng, 2013; Parkinson et al., 2011). In cultured astrocytes (but not neurons), it is predominantly metabolized to adenosine monophosphate (AMP) by adenosine kinase, and further via adenosine diphosphate (ADP) to ATP (Matz & Hertz, 1989). In adult brain, adenosine kinase is also almost selectively localized in astrocytes (Studer et al., 2006), with one of its two splice variant, the short form, in the cytosol (Boison, 2013). We have recently shown in cells freshly obtained from the mouse brain that mRNA for the mainly intracellular nucleoside transporter equilibrative nucleoside transporter 3 (ENT3; Baldwin et al., 2005), determined by reverse transcription-polymerase chain reaction (RT-PCR), is greatly enriched in astrocytes when compared with neurons. ENT3 gene expression has also previously been identified in freshly isolated astrocytes by microarray techniques (Cahoy et al., 2008; Doyle et al., 2008), and we have demonstrated its expression in cultured astrocytes by RT-PCR (Li et al., 2013). These observations lead to the suggestion that it might carry adenosine to vesicles involved in the synthesis of transmitter ATP, which in that case would be synthesized locally (Li et al., 2013). To obtain further information whether this might be the case, we have in the present study compared glutamate, adenosine, and K^+^ effects on astrocyte cultures with downregulated ENT3 due to small interfering RNA (siRNA) treatment with those in ENT-expressing astrocytes that had been treated with the other reagents needed for downregulation with siRNA, but without the ENT3 siRNA itself and were regarded as normal controls. In normal astrocyte cultures, each of these stimuli cause a large increase in ATP release (Xu et al., 2014). Furthermore, as the vesicular transporter SLC17A9 which transports ATP itself into neuronal synaptic vesicles (Larsson et al., 2012) has been demonstrated in cultured astrocytes of unknown age and differentiation (Oya et al., 2013), we have examined the postnatal development of the expression of this transporter in our well-differentiated astrocyte cultures.

## Materials and Methods

### Reagents

Chemicals for the preparation of medium and most other chemicals, including adenosine (9 -β-D-ribofuranosyladenine), glutamate (L-2-aminopentanedioic acid), and the ecto-ATPase inhibitor, ARL67156 (6 -*N,N*-diethyl-D-beta-gamma-dibromomethylene ATP), were purchased from Sigma (St. Louis, MO, USA). Deoxyribonucleotide triphosphates were obtained from TaKaRa Biotechnology (Dalian, China). Opti-MEMI and Oligofectamine used for preparation of cells with downregulated ENT3 due to treatment with siRNA against this transporter were purchased from Invitrogen (Carlsbad, CA, USA). Santa Cruz Biotechnology (Santa Cruz, CA, USA) supplied ENT3 siRNA: sc-60588. The ENLITEN ATP Assay System Bioluminescence Detection Kit was purchased from Promega (Madison, WI, USA). Primers for ENT3, SLC17A, and TATA box-binding protein (TBP) were synthesized by Sangon Co., Ltd. (Shanghai, China).

### Cell Culture

Primary cultures of astrocytes were prepared from the neopallia of the cerebral hemispheres as previously described (Juurlink & Hertz, 1992) with minor modifications (Xu et al., 2013) and planted in 24-well plates in Dulbecco’s Minimum Essential Medium (DMEM) with 7.5 mM glucose (to allow some decline between feedings), the 5.4 mM K^+^ traditionally used in our cultures, and 20% serum, which was gradually reduced to 10% after the age of 1 week during the culturing period. After the age of 2 weeks, 0.25 mM dibutyryl cyclic AMP (dBcAMP) was included in the medium. Such cultures are highly enriched in astrocytes (>95% purity of glial fibrillary acidic protein [GFAP]) and glutamine synthetase-expressing astrocytes (Hertz, Juurlink, & Szuchet, 1985). The addition of dBcAMP at this specific stage of culturing is a crucial component of our culture preparation. Thus, expression of many astrocytic genes known to exist in adult astrocytes in the brain, for example, aralar (Lovatt et al., 2007), shows a large increase after it has been added to the culturing medium (Li, Hertz, & Peng, 2012).

### Treatment With ENT3 siRNA

To allow incorporation of siRNAs into astrocytes, 3-week-old astrocyte cultures were incubated in Dulbecco’s medium without serum for 24 hr on the day before transfection. Transfection solution contained 2 μl of Oligofectamine, 40 μl of Opti-MEMI, and 2.5 μl of siRNA (666 ng) and was added to the cultures for 8 hr. Cultures treated in this manner are indicated as siRNA(+). To control for possible effects of Oligofectamine or Opti-MEMI, they were compared with siRNA(−) control cultures, which had been treated with transfection solution in a similar manner, but without siRNA addition. After this treatment, 87.5 μl of DMEM with 37.5 μl of serum was added to the cultures. The expression of mRNA of ENT3 was measured by RT-PCR 3 days after transfection.

### Reverse Transcription-Polymerase Chain Reaction

For the determination of mRNA expression of ENT3 and SLC17A9 by RT-PCR of their mRNA expression, a cell suspension was prepared by discarding the culture medium, adding Trizol to cultures on ice and scraping the cells off the culture dish. In our hands, the classical RT-PCR gives similar results as real-time RT-PCR, and the results are more directly visible (Peng et al., 2013). The RNA pellet was precipitated with isopropyl alcohol, washed with 75% ethyl alcohol, and dissolved in 10 μl sterile, distilled water, and an aliquot was used for determination of the amount of RNA. One microgram of RNA extract was used for RT, which was initiated by 5-min incubation at 65℃ of RNA extract with Random Hexamer at a final concentration of 12.5 ng/l and deoxyribonucleotide triphosphates at a final concentration of 0.5 mM. The mixture was rapidly chilled on ice and briefly spun, and 4 μl of 5 × First-Strand Buffer, 2 μl of 0.1 M dithiothreitol, and 1 μl of RNaseOUT Recombinant RNase Inhibitor (40 U/μl) were added. After the mixture had been incubated at 42℃ for 2 min, 1 μl (200 U) of Superscript II was added, and the incubation at 42℃ continued for another 50 min. Subsequently, the reaction was inactivated by heating to 70℃ for 15 min, and the mixture was chilled and briefly centrifuged.

PCR amplification was performed in a Robocycler thermocycler with 5 µg of complementary DNA, 0.2 μM of sense or antisense nucleotides, and 0.375 U of *Taq* polymerase (TaKaRa Biotechnology). The forward primer for ENT3 was 5′ATAGCAGCGTTTACGGCCTCAC3′ and its reverse primer was 5′TCACTGGATGCTGCCAGGTC3′ (Nakagawa et al., 2008). The forward primer for SLC17A9 was 5′AGGTCATCTTGCTGTCAGCC3′ and its reverse primer was 5′AAGGATCTCTCGCTCTCCTG3′ (Oya et al., 2013). TBP was used as housekeeping gene, and its forward and reverse primers, respectively, were CCACGGACAACTGCGTTGAT and CGCTCATAGCTACTGAACTG (El Marjou, Montalescot, Buzyn, & Geny, 2000). Initially, the template was denatured by heating to 94℃ for 2 min, followed by 35 amplification cycles for ENT3 and SLC17A9, and 40 for TBP. Each cycle consisted of three periods, the first for 45 s at 94℃; the second for 45 s at 59.2℃ for ENT3, 59.8℃ for SLC17A9, and 58℃ for TBP; and the third for 90 s at 72℃. The final step was extension for 10 min at 72℃. The PCR products were separated by 1.5% agarose gel electrophoresis and captured by FluorChem 5500 (Alpha Innotech, San Leandro, CA, USA). Ratios between scanned ENT3 mRNA or SLC17A9 and TBP were determined and averaged.

### Luciferin–Luciferase ATP Assay

For the determination of the extracellular ATP, a luciferin–luciferase assay (ENLITEN ATP Assay System Bioluminescence Detection Kit, Promega) and a TECAN infinite M200 Microplate Analyzer (Lausanne, Switzerland) were used to record relative light units (RLU) as described by Liu, Sabirov, and Okada (2008). As previously described (Xu et al., 2014), the assay was performed during 60 min in DMEM without serum or pH indicator at 37℃. The ecto-ATPase inhibitor ARL67156 was present in all experiments. Then, the medium was rapidly cooled to room temperature for the ATP assay and mixed with a 100 -μl aliquot of luciferin–luciferase reagent dissolved in dilution buffer provided with the assay kit, and chemiluminescence values (RLU) were recorded as an indication of ATP content. Even in the absence of any known ATP, considerable relative high RLU readings were caused by the medium or tissue. This appeared larger than the previously measured 1,200–1,600 RLU under control conditions (Xu et al., 2014), perhaps reflecting some effects of the reagents other than siRNA itself with which the cells had been treated.

### Statistics

The statistical values of the differences between individual groups were analyzed by one-way analysis of variance followed by Fisher’s least significant difference test. The level of significance was set at *p* < .05.

## Results

**Figure 1.**
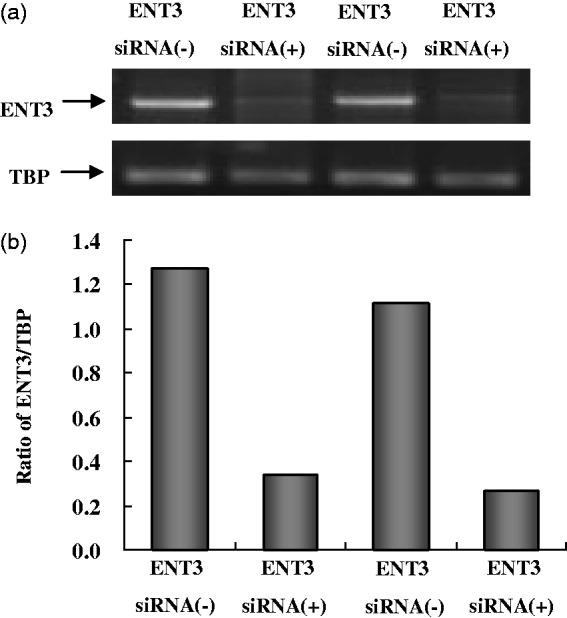
mRNA expression of ENT3 in astrocyte cultures supposed to show downregulated ENT3 expression following treatment for 3 days with transfection solution with siRNA against ENT3 [siRNA(+)], compared with cells from the same culture batch treated with transfection solution alone without siRNA against ENT3 [siRNA(−)]. mRNA expression was quantitated by RT-PCR as ratios between ENT3 and TBP, used as a housekeeping gene. (a) Southern blot from a representative experiment. (b) The size of PCR product of ENT3 is 127 bp and TBP 236 bp. The figure indicates massive downregulation of ENT3 expression only in the siRNA(+)-treated cells. *Note*. ENT3 = equilibrative nucleoside transporter 3; TBP = TATA box-binding protein; siRNA = small interfering RNA.

Figure 1(a) shows blots of two experiments in which mRNA for the ENT3 gene was determined together with mRNA for TATA-box protein, used as a housekeeping gene. In both experiments, the determination was made (a) in cultures that had undergone the whole procedure required for ENT3 downregulation by siRNA 3 days earlier [siRNA(+)] and (b) in cultures that had been exposed to the drugs used in association with siRNA treatment (Opti-MEMI and Oligofectamine) but not the siRNA itself [siRNA(−)]. The figure shows a massive ENT3 downregulation in siRNA(+) cultures but not in the cultures where the siRNA was omitted during the treatment. TBP is only slightly affected (siRNA(−) cultures). Figure 1(b) shows the scanned ratios between ENT3 and TBP mRNA expression and confirms the very considerable downregulation of the ENT3 gene by the siRNA treatment.

**Figure 2.**
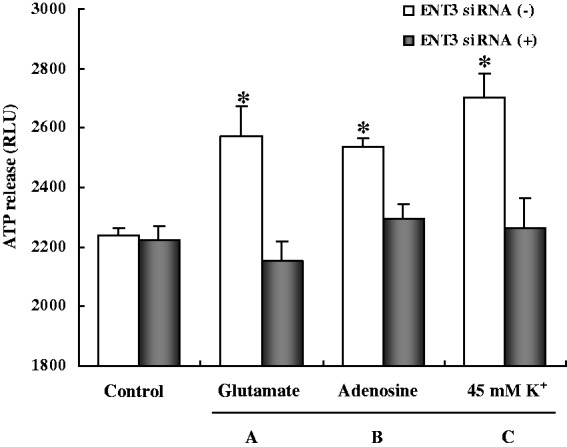
Cells were treated with glutamate, adenosine, or high K^+^ (in the presence of ARL67156). However, all experiments were carried out in either siRNA(+) or siRNA(−) cells. ATP release under control conditions was unaffected by siRNA treatment, but the response to glutamate (A), adenosine (B), and 45 mM K^+^ (C) was abolished. Results are the averages of RLU values from three to four individual cultures. SEM values are indicated by vertical bars. *Statistically significant (*p* < .05) difference from all other groups, but not each other. *Note*. RLU = relative light units; ATP = adenosine triphosphate; ENT3 = equilibrative nucleoside transporter 3; siRNA = small interfering RNA.

The ENT3 downregulation did not affect ATP release under control conditions (Figure 2), but it abolished the response to glutamate (Figure 2(A)), adenosine (Figure 2(B)), and 45 mM K^+^ (Figure 2(C)). The control values in these cultures, where even the controls had been treated with the other chemicals used during the siRNA treatment, seemed larger than previously determined (Xu et al., 2014), either reflecting larger unstimulated release or a higher blank value, and the stimulatory effects were perhaps smaller. However, the conclusion that only the cultures that had not received siRNA responded to stimulation is not affected by these possible changes.

**Figure 3.**
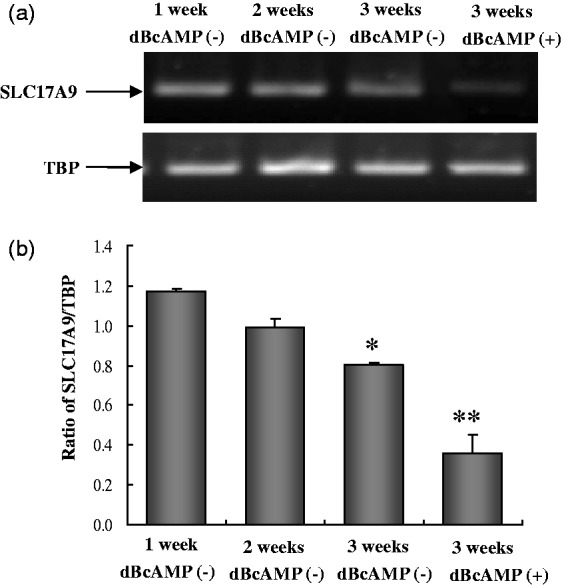
mRNA expression of SLC17A9 in astrocyte cultures at the age of 1 week, 2 weeks, 3 weeks without dBcAMP, or 3 weeks with 0.25 mM dBcAMP for the last week. The figure indicates a sharp decrease in SLC17A9 expression with the maturation of astrocytes. The size of PCR product of SLC17A9 is 189 bp and TBP 236 bp. mRNA expression was quantitated by RT-PCR as ratios between SLC17A9 and TBP from five individual cultures. SEM values are indicated by vertical bars. *Statistically significant (*p* < .05) difference from 1-week group, and **statistically significant (*p* < .05) difference from all other groups. *Note*. SLC17A9 = a neuronal vesicular transporter of ATP; dBcAMP = dibutyryl cyclic adenosine monophosphate; TBP = TATA box-binding protein; siRNA = small interfering RNA.

A rapid developmental decrease in the expression of mRNA of the vesicular ATP transporter SLC17A9 which in neurons transports ATP into synaptic vesicle is shown in Figure 3. In very immature astrocytes, its expression is pronounced, but in well-differentiated astrocytes, it has almost disappeared at the time the adenosine kinase is known to have shifted its expression from neurons to astrocytes (Studer et al., 2006).

## Discussion

The previous demonstration of the much more pronounced expression of ENT3, the only mainly intracellular nucleoside transporter (Baldwin et al., 2005), in astrocytes than in neurons (Li et al., 2013), leads to the hypothesis that the astrocytic release of transmitter ATP may originate from a vesicular ATP pool generated in situ from adenosine. This concept is supported by the present finding that downregulation of ENT3 abolishes stimulated release of ATP. In contrast, neuronal transmitter release of ATP is dependent upon an intracellular ATP transporter (Larsson et al., 2012). A similar transporter has recently been demonstrated in cultured astrocytes of unknown age and in C-6 cells, an astrocytic tumor cell line (Oya et al., 2013). However, it was associated only with lysosomes and not with other intracellular organelles containing ATP. Moreover, the age and differentiation of the astrocytic cultures are important as reflected by the virtual disappearance of the expression of this transporter in 3-week-old well-differentiated cultures shown in Figure 3. Another relevant example of differences in poorly and well-differentiated astrocyte cultures is the high rate of adenosine phosphorylation reported by Matz and Hertz (1989) in cultures similar to those used in the present study, but not by Parkinson, Ferguson, Zamzow, and Xiong (2006) in presumably less differentiated cultures. It may, however, appear against the present conclusion of adenosine transport into vesicles and intravesicular formation of ATP that Coco et al. (2003) in an article on astrocytes reported that bafilomycin A_1_ (an inhibitor of vacuolar type H^+^-ATPase [V-ATPase] involved in vesicular uptake) impaired ATP storage and release, but it should be noted that this part of their experiments was carried out using chromaffin cells, not cultured astrocytes. That mature astrocytes, in contrast to both neurons and chromaffin cells, are unable to use ATP imported by SLC17A9 for stimulated release is in support of their use of ENT3 for uptake of adenosine which is subsequently converted to ATP in the vesicles. This is also consistent with the lag time between stimulation and actual release found by both Pangrsic et al. (2007) and Pryazhnikov and Khiroug (2008).

## Summary

Glutamate, adenosine, or high K^+^ stimulation of release of gliotransmitter ATP is abolished by downregulation of adenosine transporter ENT3, probably due to the inhibition of adenosine uptake in vesicles.
